# Stepwise Total Hip Arthroplasty with Lateral and Posterolateral Approaches: Intraoperative Imaging, Fixation Strategy, and Early Functional Outcomes

**DOI:** 10.3390/life15060838

**Published:** 2025-05-22

**Authors:** Roland Fazakas, Laura Ioana Bondar, Csongor Toth, Brigitte Osser, Iosif Ilia, Gabriel Roberto Marconi, Victor Niculescu, Ramona Nicoleta Suciu, Liviu Gavrila-Ardelean, Alexandru Pop

**Affiliations:** 1 Doctoral School of Medicine, “Vasile Goldiș” Western University of Arad, 310025 Arad, Romania; fazakas.roland@uvvg.ro (R.F.); pop.alexandru@uvvg.ro (A.P.); 2 Department of Biology and Life Sciences, Faculty of Medicine, “Vasile Goldiș” Western University of Arad, 310025 Arad, Romania; 3 Doctoral School of Biomedical Sciences, University of Oradea, 410087 Oradea, Romania; csongor.toth@uav.ro (C.T.); brigitte.osser@uav.ro (B.O.); 4 Faculty of Physical Education and Sport, “Aurel Vlaicu” University of Arad, 310130 Arad, Romania; iosif.ilia@uav.ro (I.I.); gabriel.marconi@uav.ro (G.R.M.); 5 Doctoral School, University of Medicine and Pharmacy “Carol Davila” Bucuresti, 020021 București, Romania; victor.niculescu@drd.umfcd.ro; 6 Department of Psycho Neuroscience and Recovery, Faculty of Medicine and Pharmacy, University of Oradea, 410087 Oradea, Romania; ramona_suciu@uoradea.ro; 7 Prosthetic Dentistry, Faculty of Dental Medicine, “Vasile Goldiș” Western University of Arad, 310025 Arad, Romania; gavrila-ardelean.liviu@uvvg.ro; 8 Department of General Medicine, Faculty of Medicine, “Vasile Goldiș” Western University of Arad, 310025 Arad, Romania

**Keywords:** acetabular reaming, cemented hip prosthesis, intraoperative documentation, lateral approach, mid-term outcomes, patient-reported outcome measures, posterolateral approach, total hip arthroplasty, uncemented implant

## Abstract

Background/Objectives: Total hip arthroplasty (THA) remains a widely utilized and effective intervention for patients with end-stage hip osteoarthritis. Although multiple surgical approaches and fixation techniques are available, their application in non-tertiary clinical settings is less frequently documented. This study primarily aims to provide an educational overview of stepwise THA procedures using intraoperative visual documentation, with a secondary, exploratory assessment of postoperative outcomes related to surgical approach and fixation strategy. Methods: A prospective observational study was conducted at Arad Clinical Emergency County Hospital between March 2023 and March 2024, involving 23 patients undergoing primary THA. Patients received either cemented or uncemented femoral components based on intraoperative bone quality. Procedures were documented using stepwise intraoperative photographs and postoperative radiographs. Recovery was assessed using the Harris Hip Score (HHS) and the Western Ontario and McMaster Universities Osteoarthritis Index (WOMAC) at both six weeks and six months postoperatively. Results: Both lateral (Hardinge) and posterolateral approaches provided adequate exposure with reproducible results. Cemented implants allowed for immediate full weight-bearing and were preferred in elderly patients with poor bone quality, while uncemented components were used in younger patients with good bone density, requiring a delayed weight-bearing protocol. Functional scores improved in both groups between six weeks and six months. At six weeks, the mean HHS was 87.6 ± 6.2 and WOMAC 18.3 ± 4.8; by six months, these improved to 91.8 ± 5.1 and 12.7 ± 3.9, respectively. Cemented fixation demonstrated slightly better outcomes at both time points; however, intergroup differences remained below the Minimal Clinically Important Difference (MCID) thresholds. Conclusions: Tailored surgical approaches and fixation strategies, guided by intraoperative assessment, result in favorable short- and mid-term recovery profiles in THA. The integration of intraoperative visual documentation and patient-reported outcome measures (PROMs) enhances procedural transparency while supporting evidence-based decision-making and surgical training.

## 1. Introduction

Total hip arthroplasty (THA) is one of the most widely performed and successful orthopedic procedures, offering substantial improvements in pain relief, mobility, and quality of life for patients with end-stage hip disorders. Assessing functional outcomes in surgical populations, much like in sports medicine where athlete performance and recovery are systematically monitored, is essential to ensure long-term rehabilitation and overall procedural success [1,2,3,4,5].

The assessment of patient outcomes in orthopedic procedures such as THA increasingly mirrors methodologies employed across other healthcare disciplines, where treatment effectiveness is gauged through validated, patient-centered outcomes. As the global population ages and surgical indications expand, the demand for THA continues to rise, making surgical accuracy, approach selection, and implant fixation strategies increasingly important in determining long-term success [6,7,8,9,10].

Among the most debated elements of THA are the choice of surgical approach and the fixation method of the implant. Several surgical approaches are used in contemporary practice—including the direct anterior, anterolateral, lateral (Hardinge), and posterolateral techniques—each with distinct benefits and limitations in terms of exposure, muscle preservation, and dislocation risk [11,12,13]. The lateral approach offers excellent visualization and control of component placement and is particularly well-suited for elderly patients with reduced soft tissue tone [14,15,16]. In contrast, the posterolateral approach provides broader access in patients with increased adiposity or anatomic variability, though it may be associated with a higher dislocation rate if soft tissue reconstruction is insufficient [17,18].

Fixation methods are similarly tailored to patient-specific factors. Cemented prostheses remain the gold standard in elderly or osteoporotic patients due to their ability to achieve immediate mechanical fixation and reduce early implant migration [19,20,21]. Uncemented implants, which rely on bone ingrowth and hydroxyapatite-coated surfaces, are favored in younger, more active patients with adequate bone stock, offering long-term biological integration [22,23,24]. Despite their widespread use, detailed comparisons of intraoperative decision-making and implant visualization techniques remain underreported in the literature, particularly in non-tertiary hospital settings.

This study presents the operative technique and rationale used in a series of 23 patients undergoing THA at Arad County Hospital. The procedures were performed using lateral and posterolateral approaches, with both cemented and uncemented prostheses selected based on intraoperative bone quality, patient age, and anatomic considerations. In addition to standard procedural reporting, we include a series of annotated intraoperative images and radiographs to guide the reader through each step of the surgical process. Functional outcomes were assessed using patient-reported outcome measures (PROMs), including the Harris Hip Score (HHS) and the Western Ontario and McMaster Universities Osteoarthritis Index (WOMAC), evaluated at both six weeks and six months postoperatively to capture short- and mid-term recovery.

The rationale of this study is to provide an educational, visually documented account of THA, including detailed intraoperative steps and decision-making logic for surgical approach and implant selection in a general hospital setting. By combining photographic evidence, standardized surgical technique, and longitudinal functional outcomes, this article seeks to support both practicing surgeons and trainees in refining their decision-making and enhancing procedural reproducibility in everyday orthopedic practice.

This study offers several novel contributions to the existing literature. It presents the use of detailed intraoperative photographic documentation to illustrate each stage of THA in a non-tertiary, general hospital setting—an environment often under-represented in surgical reporting. It also emphasizes a pragmatic, patient-specific fixation strategy (cemented vs. uncemented), guided by real-time intraoperative assessment of bone quality. Although early functional outcomes are presented, they are intended as descriptive and exploratory findings rather than definitive comparisons. By integrating photographic evidence, PROMs, and functional outcomes within a resource-limited clinical environment, this study contributes to a more inclusive and practical understanding of THA. Furthermore, the reproducibility of both the lateral and posterolateral approaches is supported by consistent outcomes and high follow-up adherence. Together, these elements distinguish this work as both clinically relevant and educationally valuable.

## 2. Materials and Methods

### 2.1. Study Design and Setting

This prospective, observational study was conducted at the Department of Orthopedics and Traumatology, Arad Clinical Emergency County Hospital, Romania. The study included patients who underwent primary THA between March 2023 and March 2024. The procedures were performed by a single experienced surgical team using a standardized operative protocol. Intraoperative and postoperative data were collected and analyzed descriptively. Clinical documentation was supported by photographic and radiographic material obtained during the perioperative period.

### 2.2. Study Population

A total of 30 patients with advanced hip pathology were assessed for eligibility to undergo primary THA at Arad Clinical Emergency County Hospital. Each patient was evaluated preoperatively through clinical examination and radiographic assessment. Surgical indications included functional limitations, persistent pain, radiologic evidence of joint destruction, and failure of conservative management.

Eligible patients were over 50 years of age, diagnosed with advanced primary or secondary hip osteoarthritis, and scheduled for primary THA using either the lateral or posterolateral approach. Only cases with complete intraoperative photographic documentation and available postoperative radiographs were included. All participants provided written informed consent, including permission to use anonymized images for academic and research purposes.

Exclusion criteria were prior hip surgery on the affected side, planned revision THA, active infection, malignancy, severe neuromuscular disorders, or incomplete intraoperative documentation. Patients unwilling or unable to provide consent were also excluded.

**Figure 1:**
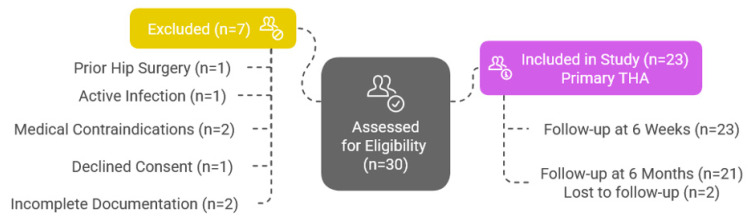
Patient selection and follow-up flowchart.

Figure 1 illustrates the patient enrollment and follow-up process. Of the 30 patients assessed, 7 were excluded: prior hip surgery (*n* = 1), active infection (*n* = 1), medical contraindications (*n* = 2), declined consent (*n* = 1), and incomplete documentation (*n* = 2). The remaining 23 patients were enrolled and underwent primary THA. Implant fixation method (cemented vs. uncemented) was determined intraoperatively based on bone quality.

Postoperative functional outcomes were assessed using the HHS and the WOMAC. All 23 patients completed the 6-week follow-up, and 21 patients (91.3%) were available at 6 months. Two patients were lost to follow-up during that interval.

This flowchart confirms the procedural consistency and strong short- to mid-term follow-up adherence, reinforcing the reliability of the outcome data.

### 2.3. Surgical Protocol

All patients were positioned in lateral decubitus. The lateral (Hardinge) or posterolateral approach (modified Hardinge/Thompson) was selected based on body habitus and surgeon discretion. Femoral neck resection was performed after joint dislocation. The acetabulum was exposed using Steinmann retractors and sequentially reamed. Trial components were tested before final implantation. Cemented implants were used in patients with osteoporotic bone, while uncemented prostheses were reserved for younger patients with good bone quality. Femoral canal preparation was performed with a progressive rasping technique. Functional testing of hip mobility was performed intraoperatively to confirm implant alignment and stability.

### 2.4. Functional Outcome Assessment

To assess patient-reported recovery, two validated PROMs were administered: the HHS and the WOMAC. These assessments were conducted at two time points: six weeks (*n* = 23) and six months postoperatively (*n* = 21). The HHS evaluates domains such as pain, function, range of motion, and deformity, while the WOMAC focuses on pain, stiffness, and physical function.

All scores were analyzed descriptively (mean ± standard deviation). To assess differences between the cemented and uncemented groups, independent samples *t*-tests were performed for both HHS and WOMAC scores at each time point. A *p*-value of <0.05 was considered statistically significant. All tests were two-tailed and based on standard formulas suitable for small-sample comparisons.

In addition to statistical significance, differences were interpreted in relation to established MCID thresholds to assess their potential clinical relevance.

### 2.5. Ethical Considerations

The study was approved by the Ethics Committee of the Arad Clinical Emergency County Hospital, Romania (protocol number 81/6 June 2024). All patients provided written informed consent for surgical treatment and use of anonymized intraoperative and radiographic images for academic and research purposes.

### 2.6. Hypotheses of the Study

This study aims to explore surgical and procedural factors influencing clinical outcomes in patients undergoing THA. Based on the current literature, institutional experience, and practical application, the following hypotheses have been formulated:

1. Association Between Surgical Approach and Intraoperative Exposure Quality: It is hypothesized that both the lateral (Hardinge) and posterolateral approaches allow for consistent and reproducible joint exposure, contributing to safe component implantation in the general hospital setting. The lateral approach is expected to offer better acetabular visualization in standard cases, while the posterolateral approach may provide advantages in patients with obesity or anatomical variations.
2. Association Between Implant Fixation Method and Early Postoperative Recovery: The study hypothesizes that implant selection tailored to patient bone quality—cemented fixation for osteoporotic bone and uncemented for younger, high-density bone—will be significantly associated with favorable early mobilization outcomes. This will be evaluated through both clinical recovery protocols and functional outcome scores, including the HHS and WOMAC. Cemented implants are expected to facilitate immediate full weight-bearing, while uncemented designs may require a protected rehabilitation protocol.
3. Educational Value and Reproducibility of Intraoperative Documentation: It is hypothesized that the integration of detailed intraoperative photographic documentation improves the educational utility of the procedure and enhances reproducibility across surgical teams and institutions. High-quality visual records are expected to serve as effective teaching aids and procedural references.

## 3. Results

The present study documents the surgical technique and intraoperative findings associated with THA performed at Arad County Hospital using both lateral (Hardinge) and posterolateral approaches. The procedure was carried out in a series of 23 patients, focusing on each key step from surgical exposure to component implantation and intraoperative stability testing. The following subsections describe each phase of the operation in detail, supported by intraoperative images and postoperative radiographic evaluation.

### 3.1. Surgical Approach

**Figure 2:**
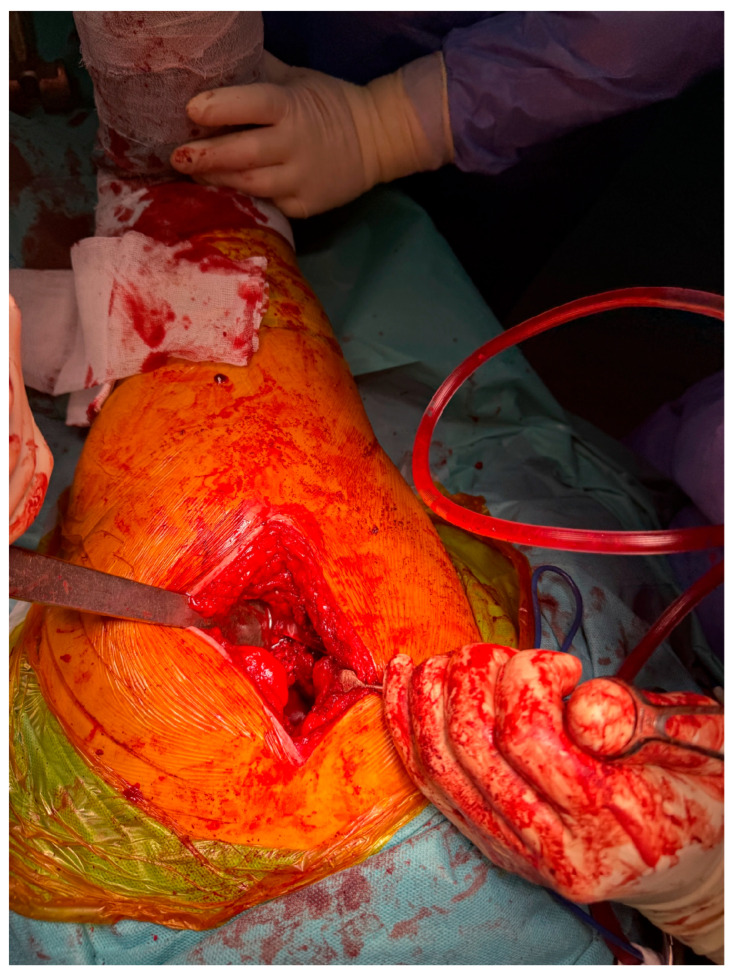
Intraoperative exposure of the femoral head and neck using the lateral (Hardinge) approach.

At Arad County Hospital, the standard technique for THA involves the lateral surgical approach, also known as the Hardinge approach. In certain cases, particularly among obese patients, the posterolateral approach—a modified Hardinge technique or the Thompson approach—is employed. The procedure begins with anatomical dissection through soft tissue planes, followed by capsulotomy. The hip joint is then dislocated, providing access to the femoral head and neck. The femoral neck is resected at an approximate angle of 45 degrees to facilitate the subsequent steps of the arthroplasty procedure (Figure 2).

**Figure 3:**
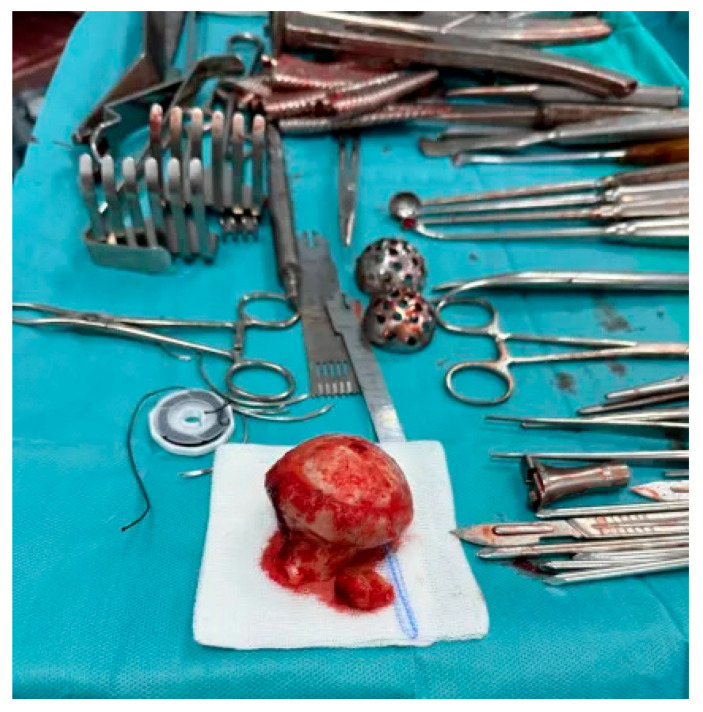
Excised femoral head affected by advanced osteoarthritis. The surface appears deformed, blunt, and flattened, resembling a melted or mushroom-like shape due to cartilage loss, subchondral sclerosis, and bone remodeling.

In advanced cases of osteoarthritis, the femoral head typically presents with deformities such as flattening, erosion, and osteophyte formation, as seen in Figure 3.

### 3.2. Acetabular Exposure and Reaming

**Figure 4:**
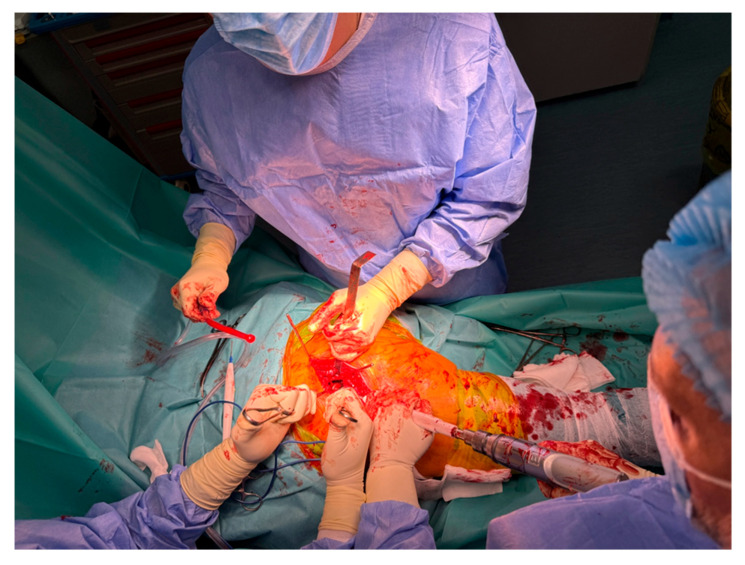
Exposure and reaming of the acetabular cavity using Steinmann retractors.

Following femoral head resection, the acetabulum is exposed using Steinmann-type retractors to provide optimal visualization of the articular cavity. A sequential reaming process is then initiated to prepare the acetabular bed. The reamers, which range in diameter from 40 mm to 60 mm, are applied progressively to achieve a concentric, hemispherical cavity of cancellous bone. This preparation is essential for ensuring proper seating of the acetabular component. Once adequate bone coverage is obtained circumferentially, trial cups are introduced to assess implant positioning, inclination, and version prior to definitive implantation (Figure 4).

### 3.3. Trial Cup Testing and Cementation

**Figure 5:**
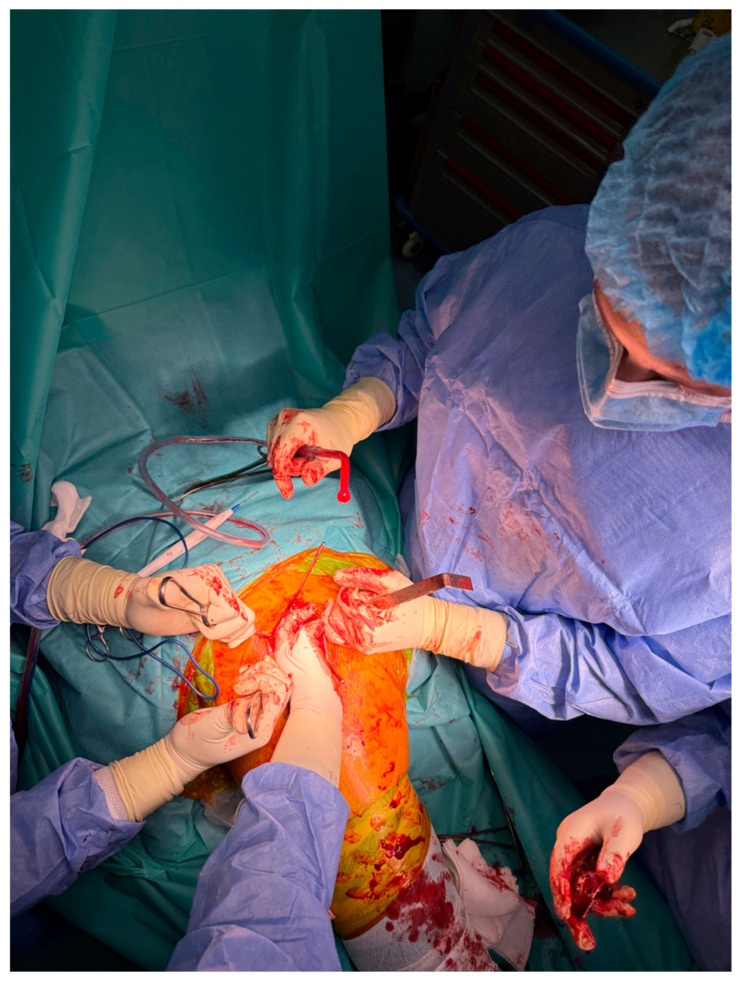
Trial cup placement to evaluate acetabular orientation.

**Figure 6:**
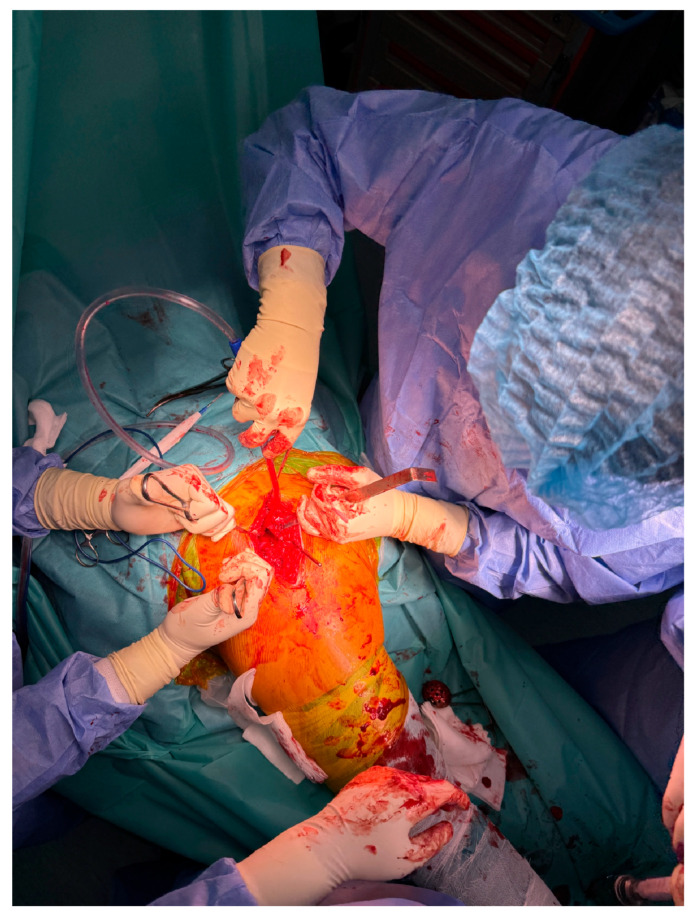
Final acetabular reaming and cemented cup fixation.

After adequate reaming of the acetabulum, trial cup testing is performed to verify correct orientation, depth, and alignment of the prosthetic component. This step is crucial to ensure that the definitive cup will achieve optimal positioning and long-term stability (Figure 5). Once satisfactory alignment is confirmed, the final acetabular cup is cemented into place using acrylic or orthopedic cement. The cement acts as a bonding interface between the polyethylene component and the prepared cancellous bone, securing the implant against micromotion and early loosening (Figure 6).

### 3.4. Femoral Canal Preparation

**Figure 7:**
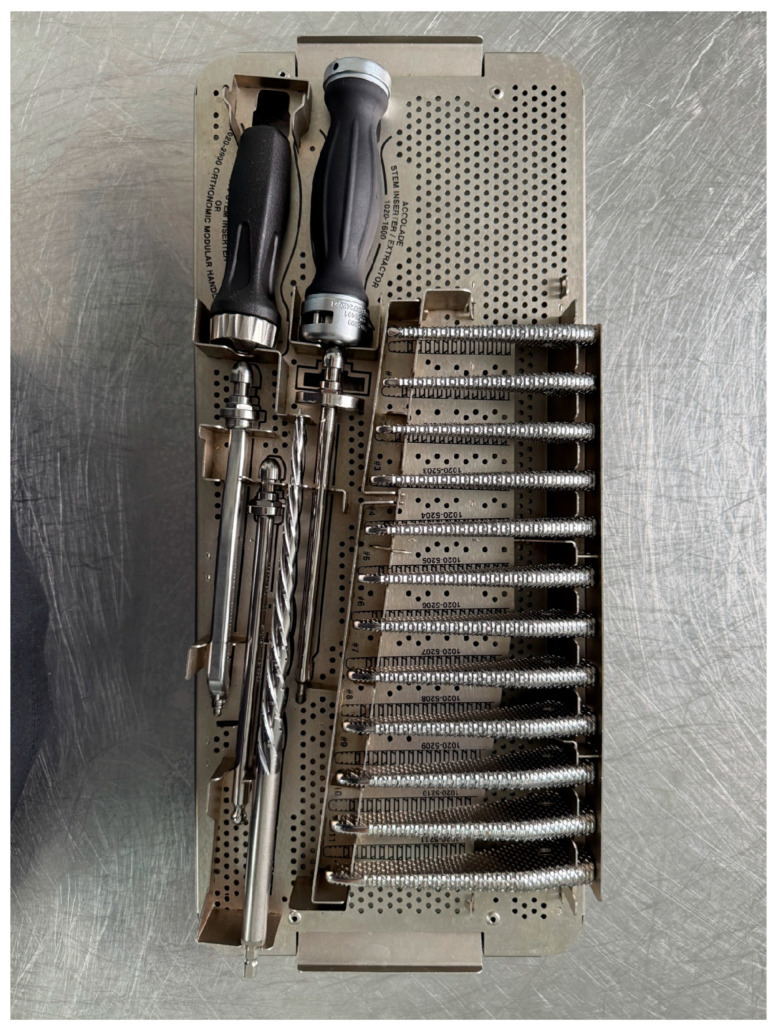
Complete set of femoral rasps arranged by size and curvature.

**Figure 8:**
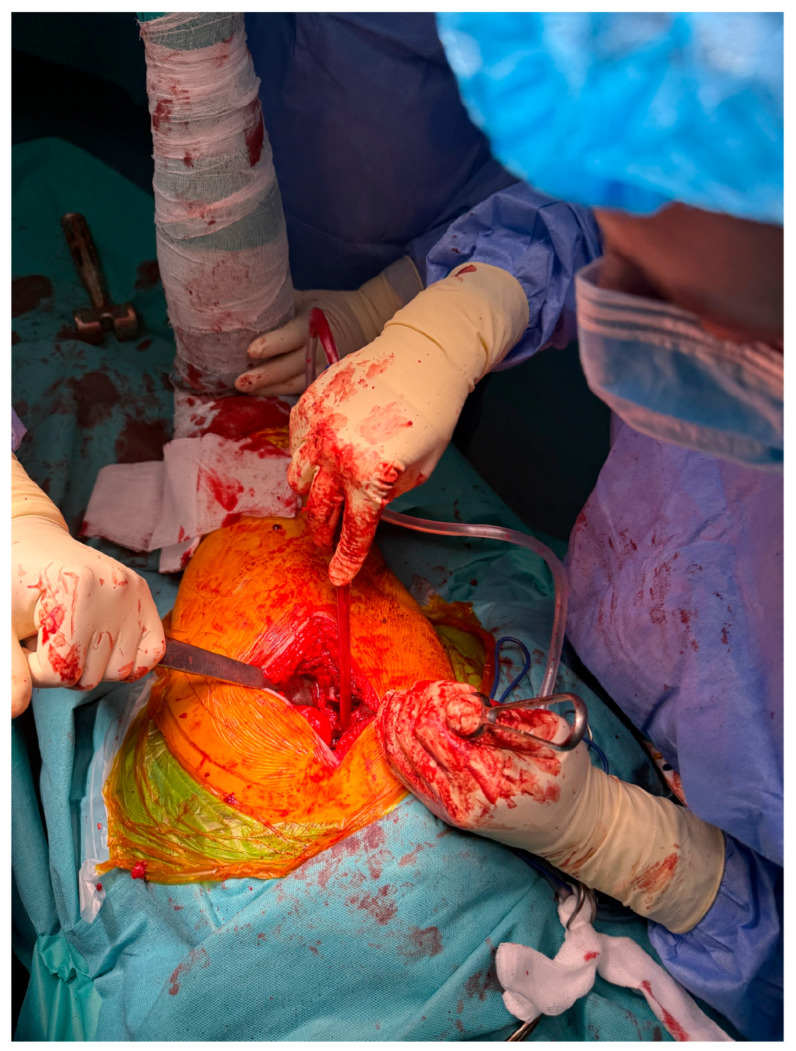
Initial exposure of the femoral shaft during THA.

Following acetabular cup implantation, attention is turned to the preparation of the femoral canal. A set of specialized femoral rasps is used in a progressive sequence to shape the canal and ensure precise fitting of the femoral component (Figure 7). These instruments are available in multiple sizes and are designed to match the geometry of the definitive implant. Prior to rasping, the femoral shaft is exposed, allowing for the correct insertion angle and depth control. The exposure also facilitates the surgeon’s visual and tactile assessment of canal integrity during instrumentation (Figure 8).

### 3.5. Progressive Rasping and Femoral Component Placement

**Figure 9:**
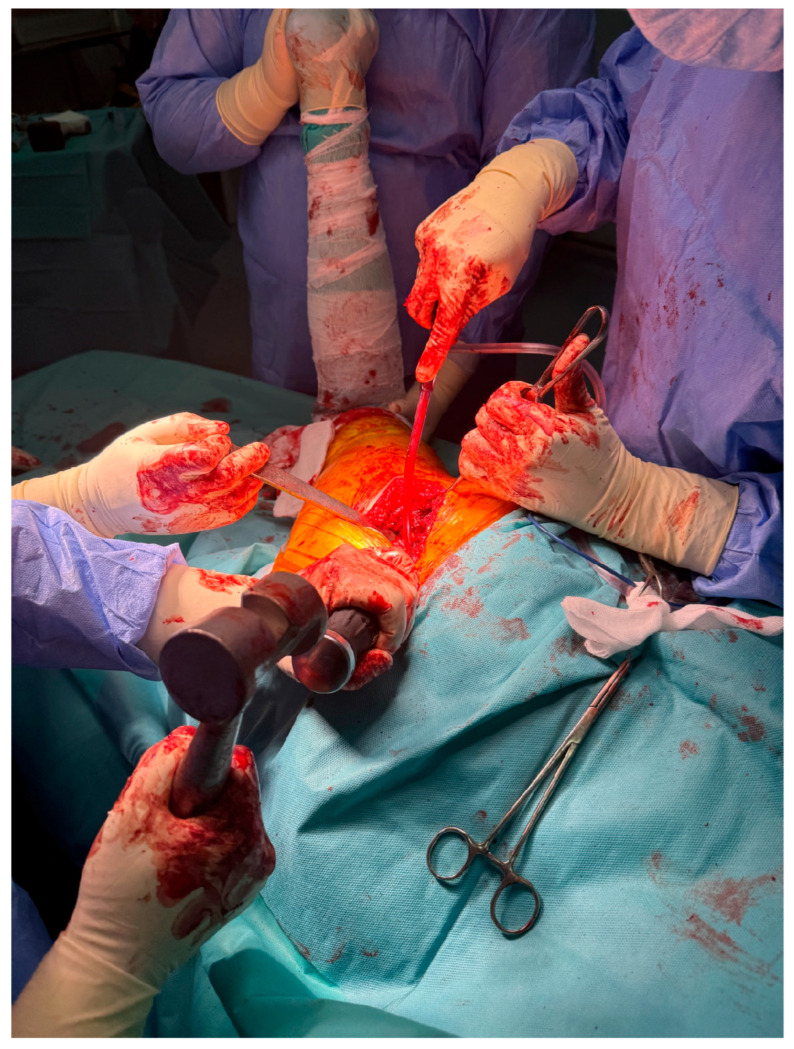
Initial stage of femoral canal rasping with early insertion of small-diameter rasp to begin shaping the medullary canal.

**Figure 10:**
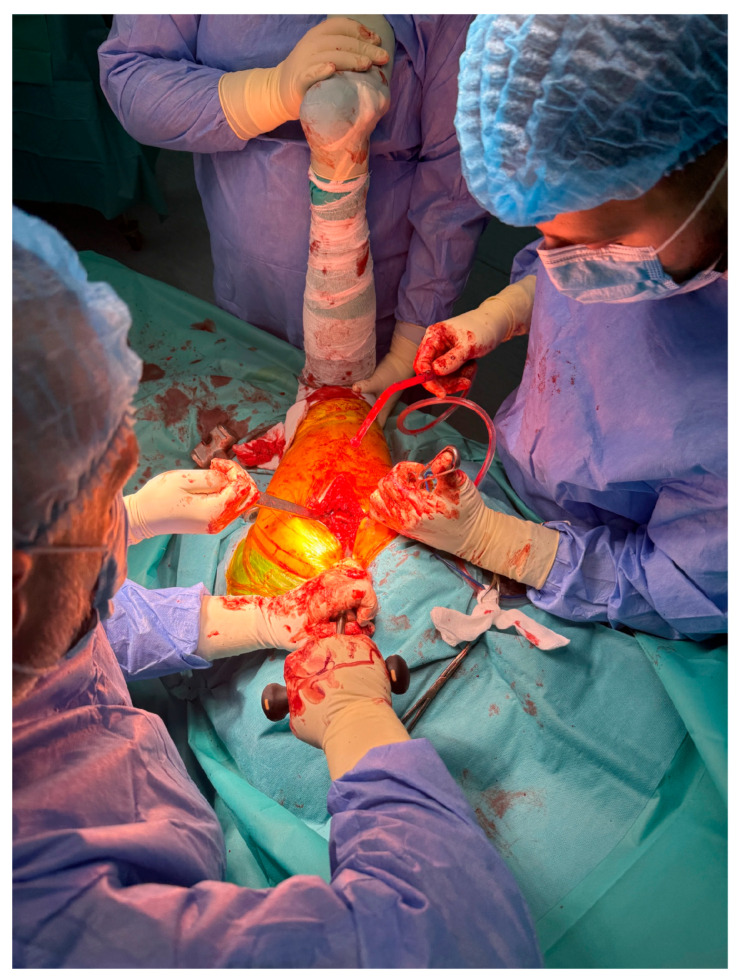
Intermediate rasping with larger implant sizer.

**Figure 11:**
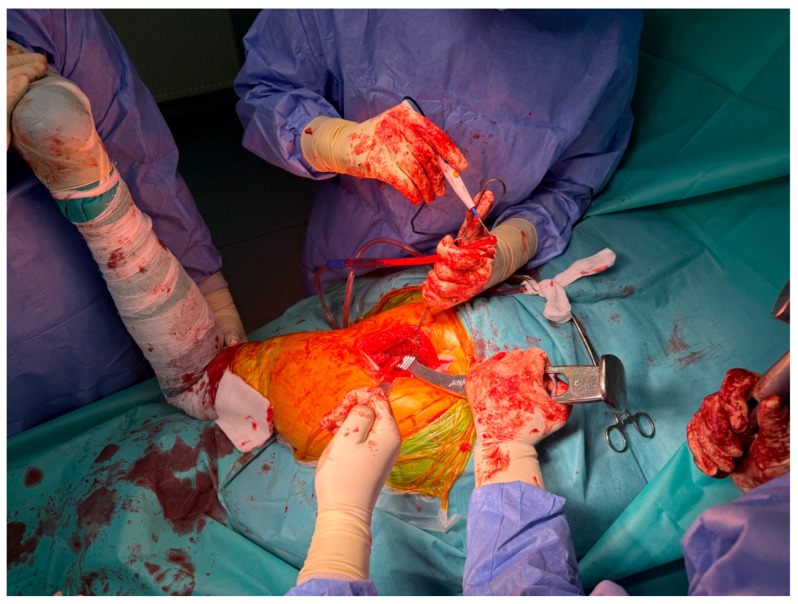
Final rasping to determine precise fit and alignment before cementing the femoral stem.

Following exposure of the femoral shaft, progressive rasping is initiated using increasingly larger rasps, beginning with size 1 and advancing until optimal canal fill, rotational stability, and approximately 10° of femoral anteversion are achieved (Figure 9, Figure 10 and Figure 11).

**Figure 12:**
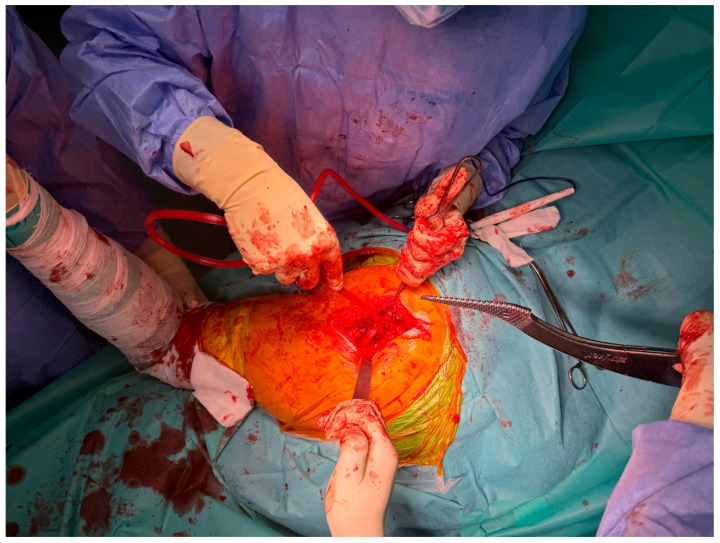
Intraoperative assessment of femoral stem anteversion and canal conformity prior to cementation.

**Figure 13:**
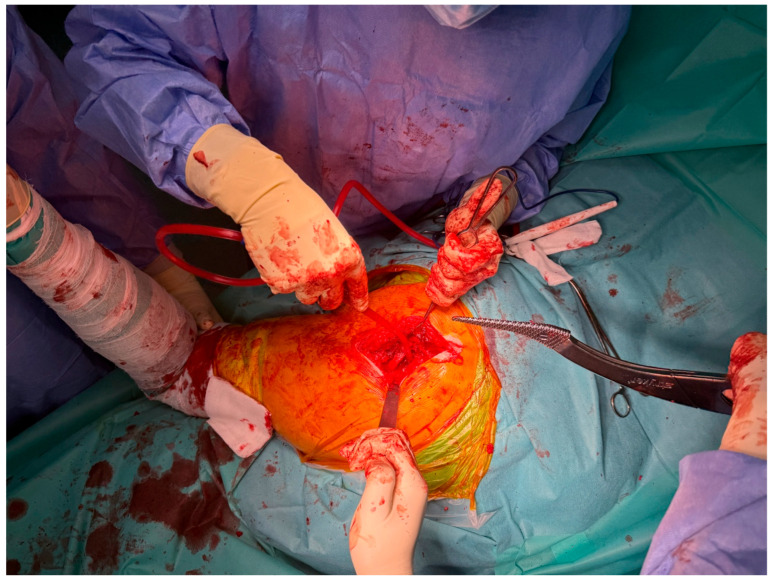
Insertion of cemented femoral component following optimal rasp selection.

Once the ideal rasp size is identified, the cemented femoral component is inserted into the prepared canal (Figure 12 and Figure 13). The prosthetic femoral head is then mounted onto the stem, completing the femoral reconstruction phase of the procedure.

### 3.6. Final Reduction and Functional Testing

**Figure 14:**
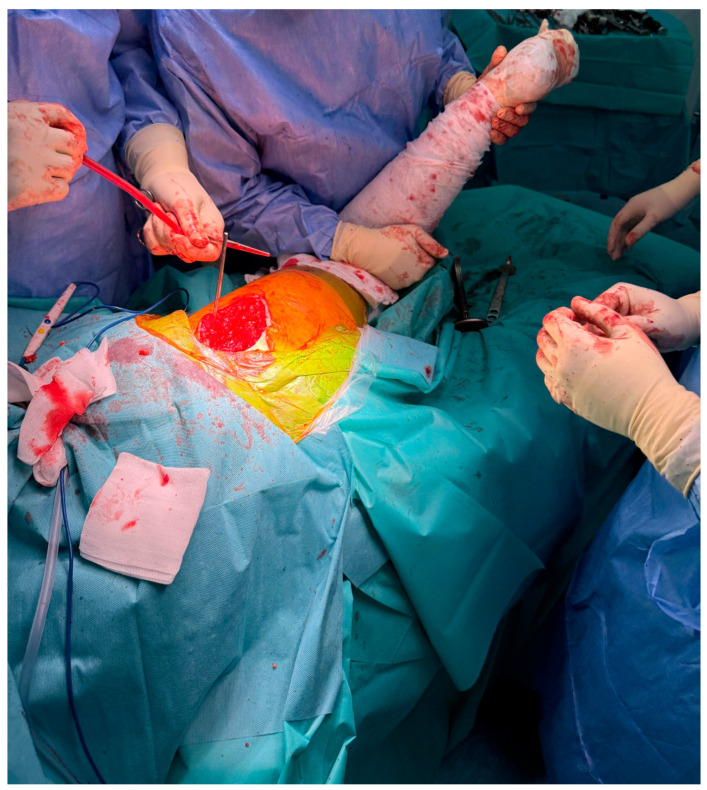
Intraoperative evaluation of joint mobility and component stability following prosthetic reduction.

Following the insertion of both the acetabular and femoral components, the hip joint is reduced to assess the functional alignment and mechanical stability of the prosthesis. The surgical team performs passive range of motion testing, including flexion, extension, adduction, and abduction, to evaluate implant articulation and soft tissue tension. This intraoperative evaluation is essential for ensuring joint congruity, confirming the absence of impingement, and verifying secure fixation before closure. Functional testing also enables the immediate identification and correction of any component malpositioning or soft tissue imbalance, which are critical for preventing early dislocation and optimizing postoperative outcomes (Figure 14).

### 3.7. Postoperative Recovery and Implant Selection

Cemented prosthetic components offer immediate mechanical fixation, enabling patients to initiate full weight-bearing ambulation as early as the first postoperative day. This characteristic is particularly beneficial for elderly patients, in whom compromised bone quality often precludes the use of press-fit techniques. Conversely, uncemented prostheses are commonly indicated in younger individuals with sufficient bone density and elasticity. These implants are typically coated with hydroxyapatite, a bioactive substance that facilitates osseointegration into the surrounding bone. This biological fixation process occurs over approximately four weeks, during which a partial weight-bearing protocol is recommended to promote proper implant stabilization and reduce the risk of micromotion.

**Figure 15:**
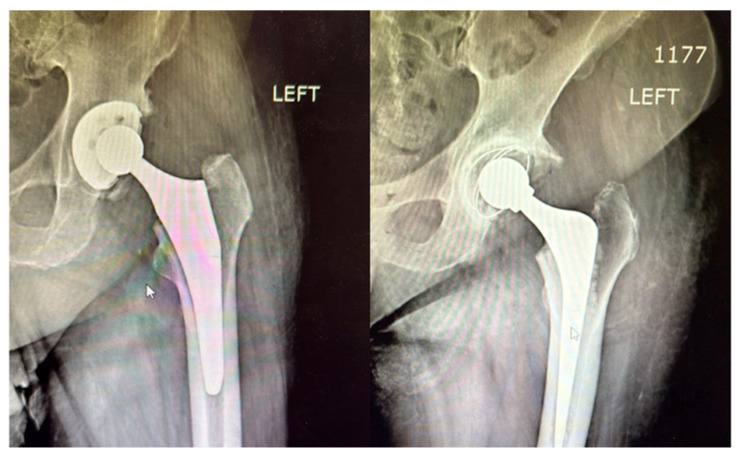
Postoperative radiographic comparison between cemented (**right**) and uncemented (**left**) femoral components in THA. The cemented stem (**right**) is outlined by a radiopaque cement mantle, while the uncemented stem (**left**) shows a press-fit alignment without visible cement, relying on bone integration for long-term stability.

The radiographic comparison shown in Figure 15 highlights the structural differences between the two types of fixations. The cemented femoral component is surrounded by a clearly visible radiopaque cement mantle, ensuring immediate stability. In contrast, the uncemented prosthesis appears press-fit within the femoral canal, relying on its surface coating and bone ingrowth for long-term fixation.

In our preliminary experience with 23 patients undergoing this surgical technique, no intraoperative complications were observed. All patients were mobilized within 24 h postoperatively, and no early signs of prosthetic loosening or malalignment were reported during the initial follow-up period. Further outcome analysis is ongoing.

### 3.8. Early Postoperative Outcomes at Six Weeks Using PROMs

As part of the study protocol, short-term patient outcomes were evaluated using validated PROMs to complement radiographic and clinical observations.

To quantitatively assess postoperative progress at six weeks, two validated PROMs were employed: the HHS and the WOMAC. These assessments were completed by all 23 patients included in the study. A summary of the collected data is presented in Table 1.

The mean postoperative HHS across the entire cohort was 87.6 ± 6.2, which is indicative of good to excellent functional outcomes. Patients who received cemented prosthetic components demonstrated a higher average score (89.2 ± 5.4) compared to those with uncemented implants (85.9 ± 6.7), suggesting a modest advantage in early mobility and function.

The mean WOMAC score, on a scale from 0 (best) to 96 (worst), was 18.3 ± 4.8, reflecting mild to moderate residual symptoms, primarily in the domains of stiffness and functional limitation. The cemented group showed slightly more favorable outcomes (16.1 ± 4.2) compared to the uncemented group (20.2 ± 5.1), consistent with the early full weight-bearing protocol typically permitted in patients receiving cemented fixation.

These findings suggest that both cemented and uncemented prostheses yielded satisfactory early results, with cemented implants demonstrating a marginally faster recovery trajectory within the first six weeks postoperatively. To assess whether these trends persisted, additional assessments were performed at six months.

Although differences in HHS between the cemented and uncemented groups at six weeks were not statistically significant (*p* = 0.2112), WOMAC scores demonstrated a borderline significant difference favoring the cemented group (*p* = 0.0496), suggesting a potential early functional benefit associated with cemented fixation (Table 2).

### 3.9. Six-Month PROMs Assessment

At six months, follow-up PROMs were available for 21 patients (91.3%). The mean HHS was 91.8 ± 5.1 (cemented: 93.2 ± 4.6; uncemented: 90.3 ± 5.4), and the WOMAC was 12.7 ± 3.9 (cemented: 11.1 ± 3.4; uncemented: 14.3 ± 4.1). Although outcomes continued to favor the cemented group, the differences between fixation types diminished over time. The intergroup differences—2.9 points for HHS and 3.2 points for WOMAC—remained below established Minimal Clinically Important Difference (MCID) thresholds, indicating that although a modest functional advantage persisted in the cemented group, it may not represent a meaningful difference from the patient’s perspective. These findings are summarized in Table 3.

Statistical analysis showed no significant differences between the groups at this time point for either HHS (*p* = 0.2041) or WOMAC (*p* = 0.0691), though a trend favoring cemented fixation remained (Table 4).

### 3.10. Visual Comparison of Patient-Reported Outcomes over Time

**Figure 16:**
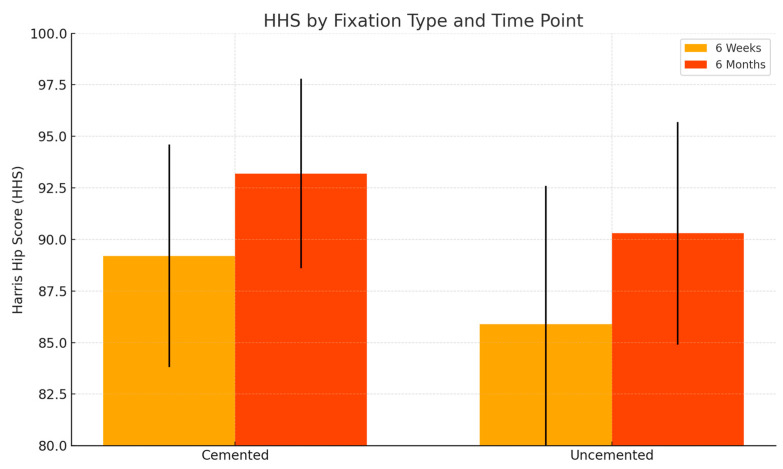
Comparison of HHS at 6 weeks and 6 months for cemented and uncemented groups.

Figure 16 visually demonstrates the progression of HHS from six weeks to six months postoperatively. Both cemented and uncemented groups demonstrated steady improvement over time. The cemented group maintained a slightly higher average score at both time points, reflecting better early recovery. However, the difference between groups narrowed at six months, suggesting that uncemented fixation approaches comparable outcomes as biological integration progresses.

**Figure 17:**
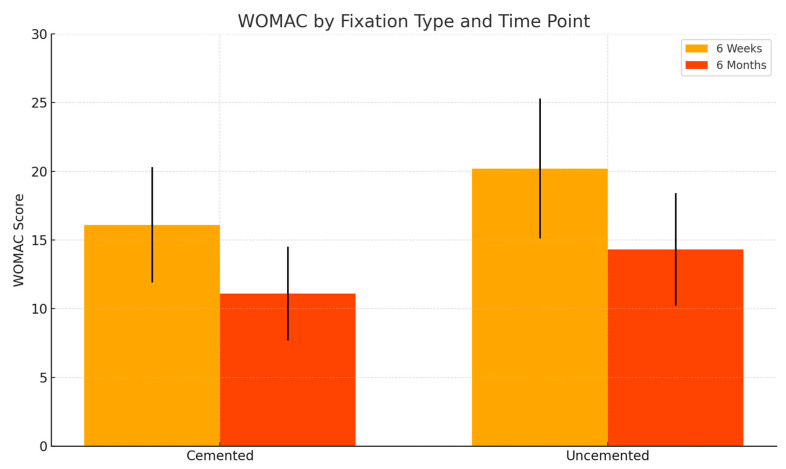
Comparison of WOMAC scores at 6 weeks and 6 months for cemented and uncemented groups.

Figure 17 visually compares WOMAC scores across both time points. Both groups experienced a reduction in WOMAC scores, indicating decreased pain and improved joint function. The cemented group showed lower scores at both time points, with a notable difference at six weeks. By six months, the scores in both groups converged, with the cemented group retaining a modest advantage. Lower WOMAC scores reflect fewer symptoms and improved joint function.

## 4. Discussion

This study examined the procedural techniques, fixation strategies, and early functional outcomes associated with THA performed using lateral and posterolateral approaches in a general hospital setting. The integration of intraoperative documentation and PROMs provided insight into surgical reproducibility and patient-centered recovery trajectories.

This study was structured around three core hypotheses: that both lateral and posterolateral surgical approaches would provide consistent intraoperative exposure; that fixation strategy tailored to bone quality would influence early postoperative recovery; and that intraoperative photographic documentation would enhance the educational utility and reproducibility of the technique. Our findings support all three hypotheses: the surgical approaches yielded adequate and reproducible exposure across all patients; cemented implants allowed for earlier functional gains, particularly in older patients, though long-term outcomes converged; and the visual documentation was successfully integrated as an instructional aid, aligning with the literature on multimedia learning in surgical education.

### 4.1. Surgical Approach and Procedural Reproducibility

This study confirmed that both the lateral (Hardinge) and posterolateral surgical approaches can be safely and reproducibly applied in a general hospital setting for THA. Among the 23 patients treated, no intraoperative complications were observed, and joint exposure was adequate in all cases. The lateral approach provided direct visualization of the acetabulum and proximal femur and was particularly effective in standard anatomical configurations. The posterolateral approach was successfully used in patients with obesity or anatomical variation, demonstrating its adaptability, especially when wider access to posterior structures was required.

These findings support the first hypothesis and align with the existing literature that recognizes the lateral approach for its stability and reduced dislocation risk, while acknowledging the posterolateral approach’s utility in complex patient morphologies [25,26,27]. Importantly, the consistency of exposure and reproducibility across all 23 cases reflects the procedural viability of these approaches even outside of high-volume arthroplasty centers.

### 4.2. Implant Fixation and Early Functional Outcomes

The study further validates the hypothesis that implant selection based on bone quality can optimize early outcomes. Cemented femoral components were used in elderly patients with osteoporotic bone, enabling immediate full weight-bearing. Uncemented implants, indicated for younger patients with preserved bone quality, required protected weight-bearing for approximately four weeks to support osseointegration.

These results are consistent with established guidelines favoring cemented stems in older adults due to immediate fixation and decreased early loosening risk [28,29,30]. The absence of early prosthetic loosening, dislocations, or malalignment during follow-up supports the effectiveness of individualized fixation strategies. Radiographic imaging confirmed the appropriate placement and stability of both cemented and uncemented components, reinforcing the clinical soundness of the fixation choices made.

### 4.3. Role of Intraoperative Documentation in Surgical Education

The final hypothesis proposed that comprehensive intraoperative documentation enhances reproducibility and serves an educational purpose. This was supported by the integration of stepwise, annotated intraoperative images in the current study, which clearly illustrate key technical stages—from exposure and reaming to trial fitting and final implantation. While many articles describe THA procedures narratively, few include real-time photographic evidence that can guide surgeons, particularly trainees, in mastering critical steps.

This visual documentation approach aligns with the current literature promoting multimedia-based learning tools in surgical education, which have been shown to improve knowledge retention and procedural confidence [31,32,33]. Moreover, the radiographic comparison of cemented versus uncemented prostheses adds an objective element to the visual presentation of technique and outcome.

### 4.4. Patient-Reported Outcomes and Early Recovery

The integration of PROMs, including the HHS and the WOMAC, provided a patient-centered framework for evaluating outcomes following THA. In this study, the mean postoperative HHS of 87.6 observed at six weeks reflects good to excellent early results, generally indicative of satisfactory progress during the initial postoperative period. By six months, scores improved further, reinforcing consistent clinical improvement in both fixation groups [34,35,36,37].

Importantly, patients who received cemented prostheses demonstrated slightly better PROMs at six weeks compared to those who received uncemented implants. This may be attributed to the immediate mechanical stability provided by cemented fixation, which facilitates early full weight-bearing and faster recovery, particularly benefiting elderly patients with reduced bone quality.

These findings underscore the importance of individualized implant selection, guided by intraoperative assessment of bone quality and patient-specific factors such as age and comorbidities. Tailored fixation strategies may enhance postoperative mobility and help reduce the risk of delayed recovery.

While radiographic evaluation remains essential for assessing implant positioning and stability, PROMs offer complementary clinical value by capturing patient-reported outcomes such as pain intensity, joint stiffness, and functional independence—factors often underrepresented in surgeon-reported metrics. Their inclusion supports a more comprehensive approach to postoperative evaluation by integrating both objective findings and patient perspectives.

It is worth noting that while the observed differences in HHS and WOMAC scores between cemented and uncemented groups were modest, they approached commonly cited thresholds for MCID. Reported MCID ranges for HHS are approximately 7–10 points, and for WOMAC, 10–12 points [38,39]. In the current study, the mean intergroup differences at six weeks—3.3 points for HHS and 4.1 for WOMAC—did not exceed these thresholds. By six months, the differences narrowed further (2.9 for HHS, 3.2 for WOMAC), reinforcing that although trends favored cemented fixation, the clinical relevance of these differences likely diminished over time.

### 4.5. Clinical Implications and Future Directions

The results of this study have several important clinical implications. First, the findings confirm that both the lateral and posterolateral approaches can be safely and effectively applied in a non-tertiary hospital setting, supporting their broader use beyond high-volume arthroplasty centers. The lateral approach may be preferred for its intraoperative stability and direct visualization of anatomical landmarks, while the posterolateral technique remains a valuable alternative for patients with obesity or complex anatomy.

Second, the tailored selection of cemented versus uncemented femoral components based on intraoperative bone quality reflects a pragmatic, patient-centered approach that aligns with current orthopedic guidelines. Cemented implants facilitated immediate weight-bearing and early discharge, which is particularly beneficial for elderly or comorbid patients. In contrast, uncemented components offered long-term biological fixation for younger patients with sufficient bone stock. Although early functional outcome trends favored cemented fixation, the modest intergroup differences did not exceed established MCID thresholds, suggesting that early recovery was comparable from the patient’s perspective.

Third, the integration of high-resolution intraoperative photographs and radiographs contributes meaningfully to surgical education. This visual documentation enhances procedural transparency and reproducibility, and may serve as a valuable teaching resource for residency programs and surgical training initiatives.

Looking ahead, increasing the sample size will be essential for assessing complication rates and long-term outcomes, including implant survivorship and delayed recovery. Further research should also expand the use of validated PROMs to monitor patient-centered recovery over longer follow-up periods. Comparative studies exploring surgical approach and fixation type across larger, more diverse cohorts will be critical for refining clinical decision-making and developing standardized protocols that can be applied across varied healthcare settings.

### 4.6. Limitations of the Study

Despite its strengths, this study is not without limitations. The relatively small sample size (*n* = 23) limits the generalizability of the findings, particularly with respect to statistical analysis of complications, subgroup comparisons, or implant survivorship. However, the primary focus of the study was descriptive and technical, and the sample was sufficient to demonstrate procedural consistency and reproducibility over a 12-month period in a real-world clinical setting.

In addition, the study design did not include randomization or a control group, which limits the strength of causal inference. Implant selection was based on intraoperative assessment of bone quality, introducing the possibility of selection bias and unmeasured confounding variables that could have influenced outcomes. These methodological limitations should be taken into account when interpreting the findings, particularly when comparing fixation groups. Additionally, as a single-surgeon, single-center study, the findings may not be fully generalizable to other institutions or surgical teams.

Additionally, although functional outcomes were assessed at both six weeks and six months using the HHS and WOMAC indices, longer-term follow-up was not available within the scope of this study. The absence of extended postoperative data limits the ability to evaluate implant longevity, complication rates, and sustained functional improvement over time.

Future follow-up of the study cohort at 12, 24, and 60 months may help address this limitation by incorporating clinical, radiographic, and patient-reported outcome evaluations, enabling a more comprehensive assessment of long-term implant performance and delayed complications.

Finally, while intraoperative photographic documentation is a key strength, it may not fully capture subtle technical nuances or soft tissue handling strategies, which often rely on the surgeon’s experience and intraoperative judgment. Nevertheless, the inclusion of detailed visual material represents an important step toward standardizing THA workflows and enhancing surgical education in general orthopedic practice.

## 5. Conclusions

This study provides a comprehensive and visually supported account of THA using both the lateral (Hardinge) and posterolateral surgical approaches, conducted over a one-year period in a real-world clinical setting. A major strength of this work is the inclusion of high-quality, step-by-step intraoperative photographic documentation (Figure 2, Figure 3, Figure 4, Figure 5, Figure 6, Figure 7, Figure 8, Figure 9, Figure 10, Figure 11, Figure 12, Figure 13 and Figure 14), which not only enhances the transparency of the surgical process but also provides significant pedagogical value. This visual material serves as a practical guide for orthopedic trainees and practicing surgeons alike, supporting surgical education and procedural standardization across diverse clinical settings.

By integrating intraoperative photographs, postoperative radiographs, and detailed procedural descriptions, the study offers both technical documentation and valuable educational content for a wide range of audiences, including surgeons, residents, and training programs. The findings support the hypothesis that both surgical approaches are safe and reproducible when appropriately selected based on patient anatomy and clinical indications. The lateral approach proved effective in most standard cases, while the posterolateral approach served as a practical alternative for patients with obesity or altered anatomical profiles.

Equally important, intraoperative decision-making regarding implant fixation—cemented versus uncemented—was shown to influence early recovery. Cemented stems provided immediate mechanical stability and allowed for early weight-bearing, particularly benefiting older patients with reduced bone quality. Uncemented prostheses, used in younger patients with adequate bone stock, demonstrated favorable outcomes through biological fixation. These results highlight the importance of patient-specific implant selection in accordance with current orthopedic best practices.

Although the sample size was limited and follow-up confined to the short- and mid-term, the study demonstrates that high-quality, consistent outcomes can be achieved in non-tertiary hospital settings when standardized protocols are applied and adapted to patient-specific factors.

Future research should aim to expand patient cohorts, extend follow-up durations, and incorporate standardized outcome scoring tools across time points. Comparative studies evaluating surgical approach and fixation method in larger populations will be essential for refining clinical decision-making and advancing surgical education in THA.

While this study suggests that both lateral and posterolateral THA approaches can be implemented safely and consistently in a general hospital environment, these findings should be interpreted in light of the study’s descriptive design. The results reflect a single-surgeon, single-center experience and may not be generalizable to all settings. Furthermore, the small sample size, lack of blinding, and limited follow-up restrict the ability to draw definitive conclusions about long-term outcomes or comparative effectiveness. Nonetheless, the integration of intraoperative visual documentation and patient-centered outcome measures supports transparency in surgical practice and provides valuable educational material for training and quality improvement efforts.

## Figures and Tables

**Table 1:** PROMs at 6 weeks postoperatively.

Outcome Measure	All Patients (*n* = 23)	Cemented Group (*n* = 12)	Uncemented Group (*n* = 11)
Harris Hip Score (Mean ± SD)	87.6 ± 6.2	89.2 ± 5.4	85.9 ± 6.7
WOMAC Score (Mean ± SD)	18.3 ± 4.8	16.1 ± 4.2	20.2 ± 5.1

Note: HHS ranges from 0 to 100, with higher scores indicating better hip function. WOMAC score ranges from 0 to 96, with lower scores representing fewer symptoms and better functional status.

**Table 2:** Statistical comparison of functional scores at 6 weeks postoperatively.

Measure	t-Value	*p*-Value
Harris Hip Score	1.293	0.2112
WOMAC Score	−2.094	0.0496

**Table 3:** PROMs at 6 months postoperatively.

Outcome Measure	All Patients (*n* = 21)	Cemented Group (*n* = 11)	Uncemented Group (*n* = 10)
HHS (Mean ± SD)	91.8 ± 5.1	93.2 ± 4.6	90.3 ± 5.4
WOMAC (Mean ± SD)	12.7 ± 3.9	11.1 ± 3.4	14.3 ± 4.1

Note: HHS ranges from 0 to 100, with higher scores indicating better hip function. WOMAC Score ranges from 0 to 96, with lower scores representing fewer symptoms and better functional status.

**Table 4:** Statistical comparison of functional scores at 6 months postoperatively.

Measure	t-Value	*p*-Value
Harris Hip Score	1.318	0.2041
WOMAC Score	−1.936	0.0691

## Data Availability

The data presented in this study are available on request from the corresponding author. The data are not publicly available due to ethical and privacy restrictions related to patient confidentiality.

## Abbreviations

The following abbreviations are used in this manuscript:

THA	Total Hip Arthroplasty
PROMs	Patient-Reported Outcome Measures
HHS	Harris Hip Score
WOMAC	Western Ontario and McMaster Universities Osteoarthritis Index
MCID	Minimal Clinically Important Difference
